# Boron neutron capture therapy outcomes for advanced or recurrent head and neck cancer

**DOI:** 10.1093/jrr/rrt098

**Published:** 2013-08-16

**Authors:** Minoru Suzuki, Ituro Kato, Teruhito Aihara, Junichi Hiratsuka, Kenichi Yoshimura, Miyuki Niimi, Yoshihiro Kimura, Yasunori Ariyoshi, Shin-ichi Haginomori, Yoshinori Sakurai, Yuko Kinashi, Shin-ichiro Masunaga, Masanori Fukushima, Koji Ono, Akira Maruhashi

**Affiliations:** 1 Particle Radiation Oncology Research Center, Research Reactor Institute, Kyoto University, 2-1010, Asashiro-nishi, Kumatori-cho, Sennan-gun, Osaka 590-0494, Japan; 2 Department of Radiation Life Science, Research Reactor Institute, Kyoto University, 2-1010, Asashiro-nishi, Kumatori-cho, Sennan-gun, Osaka 590-0494, Japan; 3 Division of Radiation Safety, Research Reactor Institute, Kyoto University, 2-1010, Asashiro-nishi, Kumatori-cho, Sennan-gun, Osaka 590-0494, Japan; 4 Department of Oral and Maxillofacial Surgery II, Graduate School of Dentistry, Osaka University, 1-8, Yamada-Oka, Suita, Osaka 565-0871, Japan; 5 Department of Otolaryngology and Head and Neck Surgery, Kawasaki Medical School, 577, Matsushima, Kurashiki-City, Okayama 701-0192, Japan; 6 Department of Radiation Oncology, Kawasaki Medical School, 577 Matsushima, Kurashiki-City, Okayama 701-0192, Japan; 7 Department of Clinical Trial Design and Management, Translational Research Center, Kyoto University Hospital, 54 Kawaracho, Shogoin, Sakyo-ku, Kyoto 606-6507, Japan; 8 Department of Dentistry and Oral Surgery, Osaka Medical College, 2-7, Daigaku-machi, Takatsuki Ciy, Osaka 569-8686, Japan; 9 Department of Otolaryngology, Osaka Medical College, 2-7, Daigaku-machi, Takatsuki City, Osaka 569-8686, Japan

**Keywords:** boron neutron capture therapy, head and neck tumors

## Abstract

We retrospectively review outcomes of applying boron neutron capture therapy (BNCT) to unresectable advanced or recurrent head and neck cancers. Patients who were treated with BNCT for either local recurrent or newly diagnosed unresectable head or neck cancers between December 2001 and September 2007 were included. Clinicopathological characteristics and clinical outcomes were retrieved from hospital records. Either a combination of borocaptate sodium and boronophenylalanine (BPA) or BPA alone were used as boron compounds. In all the treatment cases, the dose constraint was set to deliver a dose <10–12 Gy-eq to the skin or oral mucosa. There was a patient cohort of 62, with a median follow-up of 18.7 months (range, 0.7–40.8). A total of 87 BNCT procedures were performed. The overall response rate was 58% within 6 months after BNCT. The median survival time was 10.1 months from the time of BNCT. The 1- and 2-year overall survival (OS) rates were 43.1% and 24.2%, respectively. The major acute Grade 3 or 4 toxicities were hyperamylasemia (38.6%), fatigue (6.5%), mucositis/stomatitis (9.7%) and pain (9.7%), all of which were manageable. Three patients died of treatment-related toxicity. Three patients experienced carotid artery hemorrhage, two of whom had coexistent infection of the carotid artery. This study confirmed the feasibility of our dose-estimation method and that controlled trials are warranted.

## INTRODUCTION

The incidence rate in Japan of head and neck cancer during 2008 was 16.3/100 000 population, and approximately 7800 cases died during 2011. Both the incidence and mortality rates of head and neck cancer are increasing. Aggressive and combined local treatment including surgery and chemoradiation has been applied to advanced head and neck cancer, because the prognosis for patients with recurrent disease is generally poor. Unfortunately, the rate of local recurrence has been reported at 20–57% following aggressive local treatment.

For unresectable recurrent head and neck cancer, chemotherapy alone has been considered standard treatment. However, the therapeutic effect of chemotherapy is limited to palliative settings. Reirradiation with or without chemotherapy has been investigated since the 1990s as curative treatment for unresectable advanced or recurrent head and neck cancer. Single or multicenter studies of combined treatment with reirradiation and chemotherapy have demonstrated a small number of patients with long-term survival [1, 2]. Although substantial morbidity related to reirradiation has been reported in these studies, reirradiation with or without chemotherapy has the potential to cure unresectable recurrent head and neck cancer.

The rationale for application of reirradiation with boron neutron capture therapy (BNCT) for recurrent head and neck cancer is based on the unique property of BNCT, which can deposit a large dose gradient between the tumor and surrounding normal tissues. BNCT is based on the following nuclear reaction. Nonradioactive isotope ^10^B atoms that absorb low-energy (<0.5 eV) neutrons (thermal neutrons) disintegrate into an alpha (^4^He) particle and a recoiled lithium nucleus (^7^Li). These particles deposit high energy along their very short path (<10 µm) [3]. Thus, only malignant cells with ^10^B are destroyed following thermal neutron irradiation. Theoretically, any normal cells abutting the cancer cells are spared from high linear energy transfer (LET) irradiation by ^4^He and ^7^Li particles.

In 2001, a patient with recurrent parotid gland tumor after standard therapies including surgery, radiotherapy and chemotherapy was referred to the Kyoto University Research Reactor Institute (KURRI) from Osaka University, Graduate School of Dentistry. The patient was treated with BNCT at KURRI, which was the first such attempt worldwide [4]. In the first case, locoregional control of the patient was achieved for 7 years until the patient died of intercurrent disease. This promising initial result prompted clinical trials of BNCT for head and neck cancer in Japan and Finland, and several case reports of BNCT for recurrent head and neck tumors have been published by our research group [5, 6].

To investigate the efficacy and safety of applying BNCT to head and neck cancers, we conducted an outcomes study using the records of consecutive patients who were treated with BNCT.

## MATERIALS AND METHODS

### Patients

Patients who were treated with BNCT at KURRI for either locoregionally recurrent or newly diagnosed head or neck cancers between December 2001 and September 2007 were identified in the hospital's medical records. Clinicopathological characteristics and clinical outcomes of the patients were retrieved from these records. This study was performed according to Ethical Guidelines for Epidemiological Research by the Japanese Government. Informed consent was obtained according to the guidelines. The study protocol was approved by the Ethical Review Board of each medical institute.

### Overview of BNCT for head and neck tumors

Boronophenylalanine (BPA) and borocaptate sodium (BSH), which have been employed in clinical BNCT trials for malignant glioma or melanoma, were used in the present study of BNCT for head and neck cancers. All the patients showed good accumulation of BPA in the tumor in an ^18^F-BPA-positron emission tomography (PET) study before BNCT. In 72 cases, BPA in fructose solution (BPA-f) was intravenously administered at a dose of 250 or 500 mg/kg. A further 15 cases received both BSH (5 g/body) dissolved in 50% physiological saline solution and BPA-f (250 mg/kg) intravenously. Two different treatment schedules were adopted in the cases treated with BPA alone. Until May 2004, BPA was administered at a dose of 250 or 500 mg/kg in 1–2 h, followed by epithermal neutron irradiation within 15 min after finishing administration of the BPA-f solution. From June 2004, BPA was administered at a dose of 500 mg/kg in 3 h at a rate of 200 mg/kg/h for the initial 2 h, and at the reduced speed of 100 mg/kg/h for the remaining 1 h. Epithermal neutron irradiation was carried out during the final 1 h during infusion of BPA at a speed of 100 mg/kg/h. In cases administered both BSH and BPA, the epithermal neutron irradiation was started 12 h and 1 h after finishing of administration of BSH and BPA, respectively. The irradiation time was determined so that the maximum dose to the surrounding normal tissues (oral mucosa in the majority of the cases and skin in the remainder) would be <10–12 Gy-eq.

### Radiation treatment planning

Details of the procedures for radiation treatment planning with a Simulation Environment for Radiotherapy Applications (SERA) system and the Japan Atomic Energy Research Institute's Computational Dosimetry System, which are currently available BNCT treatment-planning systems, have been published [7].

SERA has been described in our previous report on a treatment-planning study of BNCT for multiple liver tumors [8]. We describe the procedure briefly here. First, the computed tomography (CT) images of each patient with head and neck tumors were inputted to the SERA system. On each slice of the CT image, the volume for the gross tumor volume and surrounding normal tissues including muscle, adipose tissue, skin and mucosa, bone and air (e.g. oral cavity and airway) were delineated. The behavior of thermal neutrons in the body is heavily affected by the proton density in the tissues; therefore, bone and air should be depicted separately.

Biological effects of boron compounds depend on their microdistribution in the tissues and the morphological characteristics of the target cells. Therefore, compound biological effectiveness (CBE) factors were used as alternative relative biological effectiveness (RBE) factors [3]. BNCT consists of mixed radiation fields, with three different types of radiation as follows: (i) low-LET γ rays, resulting primarily from the capture of thermal neutrons by normal tissue hydrogen atoms [^1^H(n,γ)^2^H] and contaminating γ-rays from the neutron beam port (bismuth-surface), the collimator and the irradiation room wall; (ii) high-LET protons, produced by the scattering of fast neutrons [^1^H(n,n)^1^H] and from the capture of thermal neutrons by nitrogen atoms [^14^N(n,p)^14^C]; and (iii) high-LET, heavier-charged particles consisting of ^4^He nuclei and ^7^Li ions, released as products of thermal neutron capture reactions (BNCR) with ^10^B [^10^B(n,α)^7^Li]. The doses by epithermal neutron beam in the absence of ^10^B comprise the low-LET γ-ray dose plus the high-LET proton dose as described above. The CBE factor was calculated using the following equation.

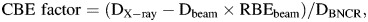

where D_X-ray,_ D_beam_ and D_BNCR_ are the doses of the reference X-ray, the epithermal neutron beam, and the ^4^He nuclei and ^7^Li particles derived from the ^10^B(n,α)^7^Li reaction required for equal biological effect; RBE_beam_ is the RBE for the epithermal neutron beam alone in the absence of ^10^B. The RBE or CBE factors for the tumor and normal tissues are summarized in Table 1.

**Table 1.** RBE and CBE factors used for conversion of physical dose (Gy) to photon-equivalent dose (Gy-eq)

BNCT dose components	Tumor	Skin	Oral mucosa
^10^B (n, α) ^7^Li	3.8 (CBE _for BPA_)	2.5 (CBE _for BPA_)	4.9 (CBE _for BPA_)
	2.5 (CBE _for BSH_)	0.8 (CBE _for BSH_)	0.3 (CBE _for BSH_)
^14^N (n, p) ^14^C	3.0	3.0	3.0
Fast neutron	3.0	3.0	3.0
gamma-ray	1.0	1.0	1.0

BNCT = boron neutron capture therapy, RBE = relative biological effectiveness, CBE = compound biological effectiveness, BPA = boronophenylalanine, BSH = borocaptate sodium.

**Table 2.** Patient and tumor characteristics

	*n*
Total cases	62
Disease presentation	
Recurrent tumor	49
Newly diagnosed tumor	13
Gender	
Male	39
Female	23
Median age (range)	61 (31–85)
Treatment sites	
Oral cavity	24
Nasal cavity, Paranasal sinuses	17
Oropharynx	5
Larynx	1
Parotid gland	3
Temporal	2
Orbit	3
Mandible	3
Neck	10
Submental	1
Histology	
Squamous	33
Mucoepidermoid	5
Adenoidcystic	4
Acinic cell carcinoma	1
Polymorphous low-grade adenocarcinoma	1
Papillary cystadenocarcinoma	1
Malignant melanoma	11
Papillary adenocarcinoma	2
Inflammatory myofibroblastic tumor	1
Undifferenfiated carcinoma	1
Angiosarcoma	1
Osteosarcoma	1

The parameters and values required in the calculation with SERA include the ^10^B concentrations in the tumor or normal tissues, the thermal neutron fluence, the nitrogen composition of the tissues, the RBE of each component of the beam, and the CBE factors of the boron compound. The accumulation of BPA in the tumor and normal tissue was imaged and quantified as a tumor/blood ratio (T/B ratio) by an ^18^F-BPA positron emission tomography (PET) study before BNCT, as previously described [4]. In the ^18^F-BPA PET study performed before BNCT, ^18^F-BPA was injected through the same route as for BNCT on the day of treatment. ^10^B concentrations in the tumor during irradiation were estimated by multiplying the T/B ratio by ^10^B concentrations in the normal tissue during irradiation. In the present study, ^10^B concentrations in the normal tissue were assumed to be equal to blood ^10^B concentrations during irradiation. ^10^B concentrations in the blood during irradiation were calculated as the mean ^10^B concentrations in the blood sampled just before and just after the irradiation, because ^10^B concentrations in blood should have been decreased after the finish of injection of BPA.

Thermal neutron fluence was measured by radioactivation of gold wires (0.25 mm in diameter and 1.0 cm long) placed on the skin surface of the lesion. The SERA system was run for the dose calculation after all the parameters were entered. The dose–volume histogram (DVH) parameters, as well as the maximum, mean and minimum doses given to the gross tumor volume, were evaluated for each case.

### Evaluation of efficacy and safety

Tumor response evaluations were performed with either CT or magnetic resonance imaging (MRI) performed within 6 months after BNCT using the RECIST (Response Evaluation Criteria in Solid Tumors) criteria version 1.0. Overall survival (OS) time was calculated from the initiation of BNCT to the date of any cause of death or last confirmed day of survival, whichever occurred first. Progression-free survival (PFS) time was calculated from the initiation of BNCT to the progression or death from any cause, whichever occurred first. Adverse events were graded according to the Common Terminology Criteria for Adverse Events v3.0 (CTCAE v3.0). Following BNCT, 40% of patients received other therapies; therefore, adverse effects that occurred within 1 month after BNCT were analyzed.

### Data management and statistics

Data collection, management and analyses were conducted by the independent datacenter in the Translational Research Center, Kyoto University Hospital. OS and PFS were analyzed using the Kaplan–Meier method. All statistical analyses were performed using SAS version 9.1.3 (SAS Institute Inc., Cary, NC, USA).

## RESULTS

### Patient characteristics

Patient characteristics are detailed in Table 2. A total of 62 patients were treated with BNCT between December 2001 and September 2007. All the patients in this study had unresectable advanced or recurrent head and neck cancers. Of the 62, 13 (21%) patients had newly diagnosed unresectable tumors, and 49 (79%) patients had recurrent tumors. In the 49 patients, 41 (84%), 36 (73%) and 39 (80%) had undergone previous surgical resection, radiotherapy and chemotherapy, respectively. The majority of treatment sites were oral cavity, nasal cavity, paranasal sinuses, or neck. Patients with squamous cell carcinoma, adenocarcinoma and malignant melanoma numbered 33 (53%), 20 (32%) and 11 (18%), respectively.

### Treatment characteristics

Treatment characteristics are summarized in Table 3. Of the 62 patients, 42 received BNCT once, 17 received it twice, two received it three times, and one received it five times. A total of 87 BNCT procedures were performed on the 62 patients. Both BSH and BPA-f were used in 13 treatments, and BPA-f alone was used in 72. In the 72 treatments with BPA-f alone, a dose of 250 mg/kg was used in five treatments and 500 mg/kg in 67.

**Table 3.** Treatment characteristics

Total BNCT treatments	87
Once	42 cases
Twice	17 cases
Three times	2 cases
Five times	1 case
Boron compound	
Both BSH and BPA	13
BPA alone	72
250 mg/kg	5
500 mg/kg	67
	Median (range)
Maximum tumor diameter (mm)	48 mm (10–135)
Maximum tumor depth (mm)	50 mm (0–100)
T/B ratio	3.0 (1.7–6.1)
Minimum tumor dose (Gy-eq)	17.9 (4.0–44.5)
Maximum skin dose (Gy-eq)	6.9 (2.7–30.3)
Maximum oral mucus dose (Gy-eq)	10.9 (4.4–17.2)

T/B ratio = tumor/blood ratio.

The median tumor diameter and depth from the skin was 48 mm (range, 10–135 mm) and 50 mm (range, 0–100 mm), respectively. The median T/B ratio assessed before BNCT by the ^18^F-BPA PET study was 3.0 (range, 1.7–6.1). The median minimum tumor dose was 17.9 Gy-eq (range, 4.0–44.5 Gy-eq). The median maximum skin and oral mucosa doses were 6.9 Gy-eq (range, 2.7–30.3 Gy-eq) and 10.9 (range, 4.4–17.2 Gy-eq), respectively.

### Treatment results

Survival data were available for 53 patients. The median follow-up was 18.7 months (range, 0.7–40.8 months). Response to BNCT was assessed using imaging studies, CT or MRI, performed within 6 months after BNCT. In 57 patients, the response data were valid. Of the 57, 16 patients (28%) showed a complete response (CR), and 17 patients (30%) had a partial response (PR). The overall response rate (CR + PR) for all the patients, the 13 patients with newly diagnosed unrespectable tumors and the 46 with recurrent tumors was 58%, 39% and 61% within 6 months after BNCT, respectively.

**Fig. 1.**
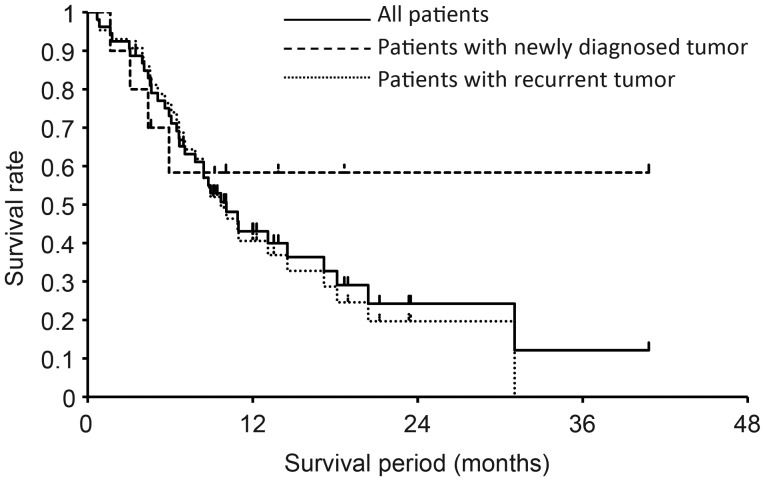
OS for all patients, those with a newly diagnosed tumor, and those with a recurrent tumor.

The median survival time (MST) was 10.1 months from the time of BNCT, and the 1- and 2-year OS rates were 43% and 24%, respectively (Fig. 1). The 1-year OS rate for the 10 patients with newly diagnosed unresectable tumors and the 43 with recurrent tumors was 58% and 41%, respectively (Fig. 1). In the patients with recurrent tumors, the MST, median PFS time and 1-year PFS rate were 9.7 months, 5.1 months and 5%, respectively. In the analysis of the patients with recurrent tumors, the 1-year OS rates for squamous cell carcinoma (*n* = 29), malignant melanoma (*n* = 6), and adenocarcinoma (*n* = 6) were 26%, 60% and 100%, respectively.

### Toxicity

The acute adverse events occurring within 1 month after BNCT were analyzed because 43% of the patients underwent other therapies following BNCT. BNCT-related acute toxicities were graded according to the CTCAE v3.0 and are listed in Table 4. The major acute Grade 3 or 4 toxicities were hyperamylasemia (38.6%), fatigue (6.5%), mucositis/stomatitis (9.7%) and pain (9.7%), all of which were manageable. One of these toxicities, hyperamylasemia, was presumably the result of irradiation of the salivary gland. This was an inevitable adverse event but did not lead to any serious situation. Three patients experienced carotid artery hemorrhage, and two died of rupture of an infected carotid artery. In addition, the carotid arteries that ruptured were invaded by recurrent tumor and reirradiated with BNCT. One patient died from malnutrition secondary to poor feeding. Details of these four patients are presented in Table 5.

**Table 4.** Acute adverse events at 1 month after BNCT

Adverse event	Grade 3	Grade 4	% Grade 3/4
Anemia	0	1	1.6
Leucopenia	0	1	1.6
Thrombocytopenia	0	1	1.6
Hyperamylasemia	10	7	27.4
Renal toxicity	1	0	1.6
Hearing loss	2	0	3.2
Otitis, external ear (non-infectious)	0	1	1.6
Otitis, middle ear (non-infectious)	1	0	1.6
Fatigue	4	0	6.5
Radiation dermatitis	1	1	3.2
Ulceration (skin)	2	0	3.2
Xerostomia	1	0	1.6
Dysphagia	2	0	3.2
Mucositis	4	2	9.7
Keratitis	1	0	1.6
Pain	6	0	9.7
Dyspnea	3	0	4.8
Edema, larynx	2	0	3.2
Vocal changes	1	0	1.6

**Table 5.** Severe BNCT-related toxicities

Patient	Treatment site	Previous RT dose	Interval between BNCT and complication (months)	BNCT tumor dose	Toxicity
1	Lt. parotid	45 Gy	18.0	66.2 Gy-eq	Lt. carotid hemorrhage requiring salvage operation
2	Lt. mandible	80.2 Gy	6.0	28.9 Gy-eq	Malnutrition due to poor feeding; patient subsequently died
3	Lt. neck	66 Gy	2.0	25.3 Gy-eq	Lt. carotid hemorrhage; patient died
4	Larynx	60 Gy	4.5	33.7 Gy-eq	Rt. carotid hemorrhage; patient died

RT = Radiotherapy.

## DISCUSSION

The purpose of this outcomes study was to investigate the safety and efficacy of BNCT for advanced or recurrent head and neck cancers. With regard to safety, the incidence of high-grade (≥3) toxicity, except for hyperamylasemia, was <10%. The incidence of Grade 3 and 4 acute dermatitis and mucositis/stomatitis was 3.2% and 9.7%, respectively, which was acceptably low considering that 79% patients with recurrent tumor received reirradiation with BNCT. A dose <10–12 Gy-eq for skin and oral mucosa was set as the dose constraint; therefore, the median maximum doses delivered to the skin and mucosa were estimated at 6.9 and 10.9 Gy-eq, respectively (Table 2). The dose calculation for BNCT has more complexity and uncertainty compared with other radiotherapy modalities. For example, the CBE factor (photon-equivalent convergent coefficient) for skin is different from that for oral mucosa, and the ^10^B concentration in normal tissues is assumed to be equal to that in the blood. Since, in this retrospective study, acute adverse effect was evaluated once at 1 month after BNCT, further prospective trials are needed for evaluation of late adverse effects. With regard to dose evaluation, acceptable acute skin and mucosa toxicity revealed in this present study suggests that the parameters and assumptions used in the BNCT dose calculation were appropriate and feasible.

One serious adverse reaction in the treatment of head and neck cancer with radiotherapy was carotid hemorrhage. This event occurred in three patients in the present study, and two of them died. The incidence rate of carotid hemorrhage (4.8%) observed in this study was comparable with that reported in the literature [9]. In all three cases, the ruptured carotid artery was invaded by the surrounding advanced tumor. Although BNCT can deposit a large dose gradient between the tumor and surrounding normal tissues, BNCT should be applied cautiously to such cases. If a carotid artery is surrounded by the tumor, angiography for evaluating the wall of the carotid artery should be performed. Irregularity of the wall suggests the invasion of the carotid artery by the surrounding tumor. BNCT should not be indicated in such cases.

The selected studies of concurrent reirradiation and chemotherapy for recurrent head and neck cancer are summarized in Table 6 [10–18]. According to these reports, concurrent chemo-reirradiation trials yielded an overall response rate ranging from 41–80%, MST 8.5–14 months, 1-year OS 37–56%, and 2-year OS 15–35%. The results in this study were difficult to compare with those in trials of chemo-irradiation as a result of the loss of survival data for 9 patients (15%), the heterogeneity of the patients and tumors, including different tumor status (recurrent or newly diagnosed), varying histology (squamous cell carcinoma, melanoma, adenocarcinoma, and others), and different chemotherapy after BNCT. However, the overall response rate and MST, and 1-year OS for recurrent head and neck cancer in the present study were 61%, 9.7 months and 41%, which are promising results and warrant a further prospective clinical trial of BNCT for recurrent head and neck cancer.

**Table 6:** Treatment outcomes of chemo-reirradiation for recurrent head and neck cancer

Authors	*n*	Radiotherapy	Chemotherapy	Response rate	Survival
De Crevoisier *et al.* [10]	169	Median 60 Gy	HU, 5-FU or MMC, 5-FU, CDDP	CR: 37%	2-year OS: 21%
				PR: 11%	
Langer *et al.* [11]	99	60 Gy: 1.5 Gy b.i.d.	CDDP, paclitaxel	Not mentioned	MST: 12.1 months
					1-year OS: 50.2%
					2-year OS: 25.9%
Spencer *et al.* [12]	52	Median 50 Gy	5-FU, HU	CR: 30%	MST: 9.4 months
				PR: 22%	1-year OS: 39%
					2-year OS: 15%
Spencer *et al.* [13]	79	60 Gy: 1.5 Gy b.i.d.	5-FU, HU	Not mentioned	MST: 8.5 months
					1-year OS: 40.5%
					2-year OS: 15.2%
Schaefer *et al.* [14]	32	Median 50 Gy	5-FU, HU	CR: 19%	MST: 9.0 months
				PR: 22%	1-year OS: 39%
Weppelmann *et al.* [15]	21	40 Gy (*n* = 11)	5FU, HU	CR: 43%	1-year OS: 56%
		48 Gy (*n* = 10)		PR: 29%	
Kramer *et al.* [16]	34	60 Gy: 1.5 Gy b.i.d.	CDDP, paclitaxel	Not mentioned	MST: 12.4 months
					1-year OS: 50%
					2-year OS: 35%
Hehr *et al.* [17]	27	40 Gy	CDDP, dicotaxel	CR: 36%	MST: 10 months
				PR: 44%	1-year OS: 37%
					3-year OS: 18%
Cohen *et al.* [18]	25	72 Gy	CDDP, tirapazamine	CR: 28%	MST: 14 months
				PR: 20%	1-year OS: 56%
					2-year OS: 27%

HU = hydroxyuria, 5-FU = 5-fluorouracil, CR = complete response, PR = partial response, OS = overall survival, b.i.d. = twice daily, CDDP = cisplatine, MST = median survival time.

Recently, intensity-modulated radiotherapy (IMRT) and stereotactic radiotherapy (SRT), as forms of reirradiation, have been applied to the treatment of recurrent, previously irradiated head and neck cancer. These techniques can deliver a definitive dose to the recurrent tumor while sparing neighboring normal tissues. Sulman *et al.* have reported the results of a study in which all 78 patients were treated with IMRT. The 2-year OS and local control rates were 58% and 64%, respectively [19]. Roh *et al.* have reported their experience of SRT using CyberKnife for recurrent head and neck cancer [20]. The response rate (CR + PR) was 80%, and the 1- and 2-year OS rates were 52.1% and 30.9%, respectively. In both studies, local control and survival data were superior to those obtained with concurrent chemo-reirradiation using conventional irradiation techniques, and treatment-related morbidity was less common. Therefore, reirradiation using these new techniques has the potential to become standard therapy for locally recurrent head and neck cancer.

In these circumstances of remarkable progress in radiotherapy techniques, we have investigated the possibility that BNCT can be applied to locally advanced or recurrent head and neck cancer. The drawback of BNCT is that deep-seated tumors cannot receive an sufficient irradiation dosethe owing to poor penetration of thermal neutrons in the body. In the present study, the maximum tumor depth ranged from 0–100 mm, and the minimum tumor dose ranged from 4.0–44.5 Gy-eq, which indicates that, in many cases, the doses delivered to the deep-seated tumors were insufficient to control the tumors. In addition, we calculated the tumor dose on the assumption that BPA distributed homogeneously in the tumor. The region with insufficient distribution of BPA may receive much a lower dose compared with the calculated minimum tumor dose. Therefore, in future clinical trials, BNCT for head and neck cancer should be limited to shallow-seated tumors, and the dose constraint should be set to the dose delivered to normal mucosa or skin in which BPA is assumed to distribute more homogeneously compared with the tumor.

All the patients with adenocarcinoma survived, although the number was very small. ^4^He particles and ^7^Li nuclei irradiating the tumor in BNCT are high-LET heavy ion particles; therefore, BNCT is theoretically expected to control radioresistant tumors such as adenocarcinoma. In addition, adenocarcinoma in the head and neck region has a tendency for microscopic perineural invasion. In IMRT or SRT for recurrent head and neck cancer, the planning target volume (PTV) margin is commonly very small (0–5 mm); therefore, microscopic perineural invasion has the risk of extending out of the PTV. In contrast, in BNCT, neutron beams irradiate the tumor with a wide margin >5 cm, using a 10–15-cm diameter round collimator. Therefore, BNCT has the potential to eradicate cancer cells that microscopically invade the tumor and exist in the perineural portion outside the gross tumor. Adenocarcinoma is a good indication for BNCT, especially when at a shallow location.

## CONCLUSION

In conclusion, this study of BNCT for advanced or recurrent tumors revealed two important findings. First, we confirmed the feasibility of our dose-evaluation method with regard to skin and oral mucosa. Second BNCT-related morbidity and mortality were acceptably low. This study warrants the clinical trial in the planning stage with full consideration of the eligibility criteria, especially in regard to the maximum tumor depth and histology.
